# Lung inflammation and genotoxicity following pulmonary exposure to nanoparticles in ApoE^-/- ^mice

**DOI:** 10.1186/1743-8977-6-2

**Published:** 2009-01-12

**Authors:** Nicklas Raun Jacobsen, Peter Møller, Keld Alstrup Jensen, Ulla Vogel, Ole Ladefoged, Steffen Loft, Håkan Wallin

**Affiliations:** 1 National Research Centre for the Working Environment, Lersø Parkallé 105, DK-2100 Copenhagen Ø, Denmark; 2 Department of Environmental Health, University of Copenhagen, Øster Farimagsgade 5A, DK-1014 Copenhagen K, Denmark; 3 Department for Toxicology and Risk Assessment, National Food Institute, Technical University of Denmark, Mørkhøj Bygade 19, DK-2860 Søborg, Denmark; 4 Institute for Science, Systems and Models, University of Roskilde, DK-4000 Roskilde, Denmark

## Abstract

**Background:**

The toxic and inflammatory potential of 5 different types of nanoparticles were studied in a sensitive model for pulmonary effects in apolipoprotein E knockout mice (ApoE^-/-^). We studied the effects instillation or inhalation Printex 90 of carbon black (CB) and compared CB instillation in ApoE-/- and C57 mice. Three and 24 h after pulmonary exposure, inflammation was assessed by mRNA levels of cytokines in lung tissue, cell composition, genotoxicity, protein and lactate dehydrogenase activity in broncho-alveolar lavage (BAL) fluid.

**Results:**

Firstly, we found that intratracheal instillation of CB caused far more pulmonary toxicity in ApoE^-/- ^mice than in C57 mice. Secondly, we showed that instillation of CB was more toxic than inhalation of a presumed similar dose with respect to inflammation in the lungs of ApoE^-/- ^mice. Thirdly, we compared effects of instillation in ApoE^-/- ^mice of three carbonaceous particles; CB, fullerenes C_60 _(C_60_) and single walled carbon nanotubes (SWCNT) as well as gold particles and quantum dots (QDs). Characterization of the instillation media revealed that all particles were delivered as agglomerates and aggregates. Significant increases in *Il-6, Mip-2 *and *Mcp-1 *mRNA were detected in lung tissue, 3 h and 24 h following instillation of SWCNT, CB and QDs. DNA damage in BAL cells, the fraction of neutrophils in BAL cells and protein in BAL fluid increased statistically significantly. Gold and C_60 _particles caused much weaker inflammatory responses.

**Conclusion:**

Our data suggest that ApoE^-/- ^model is sensitive for evaluating particle induced inflammation. Overall QDs had greatest effects followed by CB and SWCNT with C_60 _and gold being least inflammatory and DNA-damaging. However the gold was used at a much lower mass dose than the other particles. The strong effects of QDs were likely due to Cd release. The surface area of the instilled dose correlated well the inflammatory response for low toxicity particles.

## Background

Human beings have always been exposed to airborne ultrafine particles (i.e. particles below 100 nm size) from e.g., forest fires, volcanic eruptions or indoor fire places. However, since the industrial revolution, exposures to ultrafine particles have increased dramatically. This is mainly due to the invention of the combustion engines [1]. Engineered nanomaterials and nanotechnologies are expected to have a profound impact on many aspects of society and economy. However, they also represent a new source of human exposures and awareness is growing that their unusual chemical and physical properties may lead to potential environmental and health risks [1,2]. The toxicity and carcinogenicity of low-soluble particles is thought to be exerted primarily through generation of inflammation and oxidative stress [3]. Pulmonary inflammation is central in lung diseases and also thought to be involved in risk of atherothrombosis [4,5]. Concern has been raised over nano-sized particles because the bronchio-alveolar deposition rate is great, they have a large surface area, are more reactive, and the clearance of them is slow. All these factors may contribute to more severe and prolonged inflammation and, consequently, increased risk for disease. Although it has been estimated that less than 1% of the deposited dose translocates from the lung into the circulation, this is suspected to be important in nanoparticle-induced cardiovascular effects, and may also produce adverse hepatic, developmental effects and other effects in organs remote from the lung [6]. Although many laboratories are currently investigating toxicological effects of nano-sized materials, few *in vivo *studies have been published in the field on nanotoxicology and only with a very limited panels of nanomaterials in the same experimental setup.

For an *in vivo *comparison study of toxicity we chose a panel of nanoparticles with carbon black, Printex 90 (CB), single walled carbon nanotubes (SWCNT), fullerenes C_60 _(C_60_), quantum dots (QDs) and nanosized gold particles. CB is a well-known ingredient in rubber, plastics, inks, and paints with an annual production about 10 million tonnes [7]. The toxic effects of CB have been well described *in vitro *and *in vivo*, making it an excellent benchmark material. SWCNT and C_60 _have the potential to be among the most widely used carbonaceous engineered nanomaterials in the future. Material scientists have envisioned the use of these type of particles in wide range of applications (e.g., composite materials, disease treatment and electronics)[8-10]. QDs are currently applied in biomedical imaging and electronics, but have been suggested for use in computer memories, visual displays, solar cells and lasers [11] as well as a replacement for organic dyes due to superior quantum yield and resistance to photo bleaching [12]. Both QDs and gold particles additionally have properties which make them detectable in translocation and targeting studies [13-17]. The overall goal of this study was to test an expected susceptible animal model (apolipoprotein E knockout mouse, ApoE^-/-^) as well as background strain (C57) for direct comparison of markers of inflammation, lung injury and genotoxicity in lung tissue and broncho-alveolar lavage (BAL) fluid following pulmonary exposure across the panel of nanoparticles. The ApoE^-/- ^mice were chosen since these experiments were part of a larger study which also includes cardiovascular research. However, these animals have previously shown increased permeability of particulates in blood vessels [18-20]. If this permeability is related to the modest elevated blood cholesterol found in ApoE^-/- ^mice, the model may closer resemble humans with elevated cholesterol levels. Experiments showed that the most sensitive of the tested exposure methods was a combination of intratracheal (i.t.) instillation of nanoparticles in apolipoprotein E knockout mice (ApoE^-/-^). The chosen inflammatory markers were mRNA levels of macrophage inflammatory protein-2 (*Mip-2*), interleukin-6 (*Il-6*) and macrophages/monocyte chemoattractant protein-1 (*Mcp-1*) in lung tissue and BAL cell composition. MIP-2 is involved in the chemotactic recruitment of neutrophils to the pulmonary system following exposure [21,22]. MCP-1 is produced by numerous inflammatory cells including epithelium fibroblasts, monocytes and macrophages and is believed to promote the maturation of monocytes to macrophages as well as being a major chemoattractant for monocyte recruitment [23,24]. Il-6 is an important early mediator of inflammation, involved in fever and acute phase responses. It is secreted mainly by macrophages e.g. as a response in particle induced inflammation [25]. We have reported before that all of these markers are elevated at 24 h after inhalation of diesel particles [26]. Additionally, we determined DNA damage in BAL cells by the comet assay as a sensitive marker for particle genotoxicity related mainly to oxidative stress and cellular damage by leakage of protein and lactate dehydrogenase (LDH) to the BAL fluid.

## Results

### Exposure characterization

The number- and volume-size distribution of the suspended particle preparations used for i.t. instillation were characterized by dynamic light scattering (DLS) and optical microscopy, whereas the airborne CB exposure was characterized by analysis of number- and mass-size distribution.

#### Analysis of the suspended particle samples

All samples were analysed immediately after thawing and thermal acclimatization of sample aliquots in the same way as during the i.t. exposure. Reliable DLS data were not obtained for all samples, probably because of agglomeration and settling problem in the samples.

##### Vehicle (10% BAL fluid in 0.9% NaCl)

**Figure 1:**
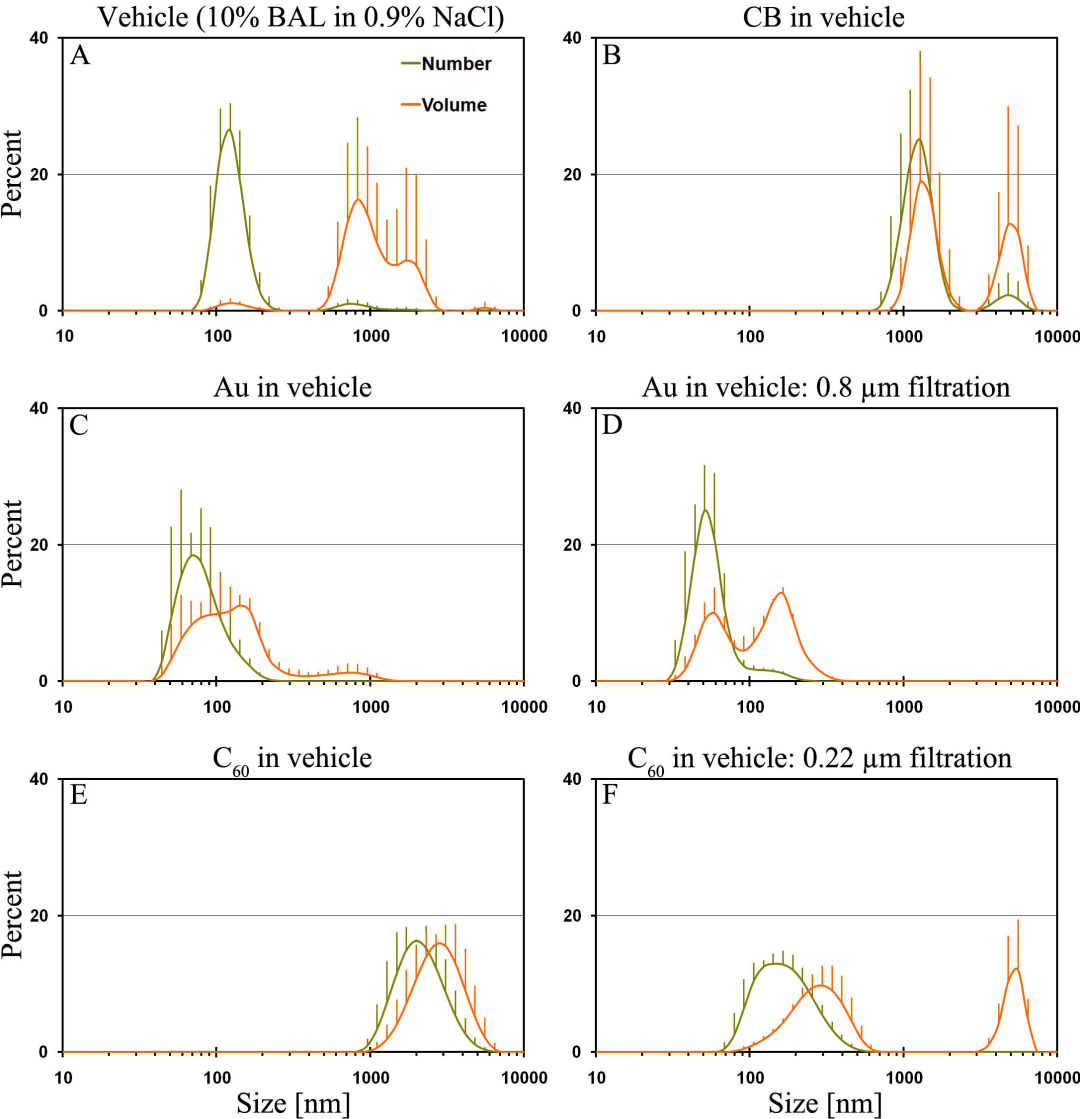
**Number and volume size distribution of the particle suspensions used for instillation determined by DLS analysis**. A) Unfiltered instillation media consisting of 0.9% NaCl added 10% BAL fluid (n = 4). B) Unfiltered stock solution of suspended CB (n = 5). C) Unfiltered stock solution of 2 nm Au particles (n = 4). D) 0.8 μm-filtered stock solution of 2 nm Au-particles (n = 4). The filtered sample show smaller particle size than the unfiltered sample and bimodal distribution by volume. E) Unfiltered stock solution of C_60 _(n = 4). F) 0.22 μm filtered C_60 _stock solution (n = 5). The filtered sample reveals the presence of 70–600 nm-size C_60 _particles not detectable in the bulk stock solution.

Analysis of the vehicle showed the presence of particles, which occur with a peak around 120 nm in the number size distribution. This may be phospholipids, proteins and smaller cellular remains. By volume the size distribution was dominated by coarser particles in the range of 0.5–3 μm, which we interpreted to be cell-fragments derived from the BAL-fluid (Fig. 1A). The cell-fragments could be disturbing factor in some of the unfiltered analysis with small particle sizes. However most of the potential disturbance appears to be sufficiently masked by the tested nanoparticles due to their higher refraction indices and light absorbing properties. As expected saline control was particle free.

##### Carbon Black

CB suspended in instillation media showed a bimodal size-distribution with one mode around 1.2 μm and a less frequent mode around 5.5 μm. The analysis was often disturbed by agglomeration indicating that the particle suspension was unstable and that coarser particles settled out. Filtering through a 3.0 μm syringe filter did not yield reliable results.

##### Gold (Au) clusters

DLS analysis of the 2 nm gold particles revealed a relatively stable suspension of agglomerated particles. The average size increased 9% over 10 min. Most particles occurred between 40 and 200 nm size. However, analysis of the volume distribution was broad and spanned from 40 nm to ~1.5 μm with the average volume zeta-size of 139.6 ± 4.5 nm. Filtering through a 0.8 μm filter confirmed the presence of a clear ~60 nm-size mode in the volume distribution and a coarser mode with a peak around 165 nm (Fig. 1D). No particles smaller than ~30 nm were detected by further filtering the suspension through a 0.22 μm filter.

##### Fullerenes C_60_

Analysis of the unfiltered fullerene suspension suggested that the majority of the particles occurred in agglomerates and aggregates (hereafter denoted agglomerates) larger than 1 μm (Fig. 1E). The average volume zeta-size was ~2 ± 0.3 μm and it increased from 1.7 to 2.4 over ~20 min, indicating agglomeration and settling during the measurements, despite that the stability criteria of the analysis were met. Filtering the suspension through 0.45 and 0.22 μm filters revealed the presence of smaller C_60 _agglomerates with a peak between 122.4 and 164.2 nm (Fig. 1F). The average zeta-size volume of the 0.22 μm filtered sample was 211 ± 14 nm; almost the same size as detected in 0.45 μm-filtered sample (257 ± 15 nm). These particles could not be detected in the unfiltered stock solution owing to masking effects by the coarse particles. The volume distribution of both the filtered samples immediately showed presence of micron-sized particles. This suggests that the particle concentration, even in the filtered samples, were too high to produce stable solutions with nano- or near nano-size C_60 _agglomerates in this medium. Presence of important amounts of individual C_60 _particles in the exposure liquids seems unlikely from these analysis.

##### Single-Walled Carbon Nanotubes

Acceptable DLS data could not be obtained for SWCNT at all. The problems of DLS analysis the SWCNT-sample may partly also be a due to the complex morphology and bundling of the SWCNT.

##### Quantum dots

It was not possible to obtain acceptable DLS data for neither the negatively charged (ADS620QD) nor the positively (ADS621QD) CdTe QDs. Adding the QDs to the instillation media resulted in an inhomogeneous solution that could not be analyzed by DLS. However, the QDs maintained their fluorescence suggesting that they still occurred as individual particles. The effect was not observed when adding QD-free vehicle with the thioglycolic acid stabilizer.

#### Size distribution of CB and deposited dose in the inhalation study

**Figure 2:**
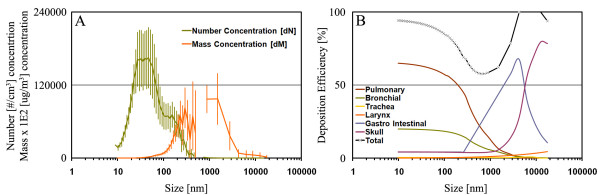
**A) The average number and mass distribution of aerosolized CB during a 1-hour experiment**. The mass concentration was calculated assuming spherical particles with a density of 2.1 μg/μm^3^. Error bars denote the standard deviation of the measured concentrations over the whole test period. B) A conservative model for deposition efficiency for particles in mice based on data from Raabe and co-workers [27]. The crosses plotted for the "Total" deposition efficiency curve indicates the model resolution, which fits the GRIMM SMPS+C and the GRIMM Dustmonitor.

The particle number- and mass-size distributions of the CB exposure are shown in Figure 2A. The number concentration and mass distributions have a 50% midpoint at 45 and 331 nm, respectively. However, the mass size distribution was widely distributed between 200 and 2750 nm (Fig. 2A). For assessment of deposited mass we developed a model based on respiratory and gastro-intestinal deposition data previously reported [27]. Unfortunately we were unable to obtain mouse deposition data for particles smaller than 270 nm. Therefore we made a conservative estimate for the deposition efficiency for smaller particles (Fig. 2B). However, it should be noted that during the inhalation experiment the majority of the mass were above 270 nm. Using this model, we estimated that 33.8% of the inhaled mass ends up in the pulmonary region. This deposition is similar to previously reported findings in mice [28], rats [16] and humans [29]. This suggests that the model is relatively precise. The model also suggests an additional deposition of 11.6% in the bronchia, 0.9% in trachea, 0.5% in larynx, 6% in the skull, and 17.1% in the gastro-intestinal tract. Hence in total 70% of the inhaled particle mass may be assumed to be deposited in the mice of which 46.8% deposits directly in the respiratory tract from the larynx to the alveoli. With air-concentration of 60 mg CB/m^3^, the total inhaled dose was 52 μg for the 1/2 h exposure and 156 μg for 1 1/2 h exposure (assuming: 240 breaths/min and 120 μl/breath). Consequently, we estimated the pulmonary deposited dose to be 17.6 and 52.7 μg, respectively, and a total respiratory tract deposition of 24.3 and 73 μg, respectively.

### Toxicity testing

#### Instillation of CB in C57 and ApoE^-/- ^mice

To detect possible differences between ApoE^-/- ^mice and the background strain C57 we instilled CB in mice of both strains and compared this to control instillations.

##### Lung tissue: Expression of mRNA of cytokines

The background levels of *Mip-2*, *Mcp-1 *and *Il-6 *were very similar in the two strains of mice as seen in the controls (Table 1). Only the *Il-6 *levels differed with levels in C57 being approximately 2-fold higher than the levels in the ApoE^-/- ^mice. In general, the exposure to CB by instillation resulted in 10- to 26-fold induction of the cytokine mRNA levels at the early time point in ApoE^-/-^, whereas C57 mice only showed between 1.5 to 2.1-fold induction compared to their controls. Following 24 h the induction was 26- to 40-fold among ApoE^-/- ^mice and 10- to 11-fold in C57 mice (strain × exposure interaction, p < 0.001 (*Mip-2 *and *Mcp-1*) and p < 0.05 (*Il-6*), ANOVA). For the *Il-6 *mRNA levels, there was also a weak interaction between the exposure and time (p < 0.05, ANOVA). The C57 mice, as compared to the ApoE^-/- ^mice strain, had lower mRNA levels of *Mcp-1 *at the 3 h time point (strain × time interaction, p < 0.05, ANOVA). This can be explained by a faster response in terms of *Mcp-1 *mRNA levels in the ApoE^-/- ^mice that resulted in 26-fold induction at the early time point, whereas the C57 mice only showed a 1.7-fold induction compared to their controls.

**Table 1:** mRNAs of *Mip-2*, *Mcp-1 *and *Il-6 *in lung tissue and cell distribution and protein in BAL fluid 3 and 24 h after instillation of carbon black in C57 and ApoE^-/- ^mice.

		**C57**	**C57**	**ApoE**^-/-^	**ApoE**^-/-^
		**Control**	**CB 54 μg**	**Control**	**CB 54 μg**
**3 h**					
**lung tissue**	***Mip-2***	9.6 ± 2.5	20.4 ± 5.9	10.2 ± 3.2	108.1 ± 15.1***
	***Mcp-1***	12.1 ± 4.6	20.6 ± 5.4	10.1 ± 1.2	265.5 ± 163.9***
	***Il-6***	4.0 ± 1.9	5.8 ± 2.3	2.1 ± 0.4	31.4 ± 4.8***
					
**BAL**	**Neutrophils%**^a^	3.4 ± 2.4	4.5 ± 0.4	3.7 ± 1.2	13.8 ± 10.9
	**Macrophages%**^a^	94.5 ± 2.6	93.8 ± 0.4	93.9 ± 2.0	83.1 ± 10.7
	**Total BAL cells**	55732 ± 14617	73407 ± 9267	83262 ± 4819	49417 ± 7700
	**Protein**	133.3 ± 21.7	82.4 ± 6.0*	102.5 ± 5.2	139.3 ± 17.3**
					
**24 h**					
**lung tissue**	***Mip-2***	7.8 ± 1.0	82.8 ± 24.8***	5.1 ± 0.5	134.8 ± 33.2***
	***Mcp-1***	39.1 ± 10.4	434.1 ± 145.8***	28.3 ± 2.7	1087.0 ± 310.6***
	***Il-6***	2.1 ± 0.6	20.3 ± 12.3*	1.1 ± 0.1	44.0 ± 13.0***
					
**BAL**	**Neutrophils%**^a^	5.2 ± 1.2	51.0 ± 12.6**	5.3 ± 1.6	75.8 ± 3.4***
	**Macrophages%**^a^	92.6 ± 2.3	47.9 ± 12.7**	93.6 ± 1.5	22.1 ± 3.7***
	**Total BAL cells**	49022 ± 3589	98857 ± 11618	65290 ± 5246	78596 ± 21414
	**Protein**	114.5 ± 13.7	124.7 ± 10.6	110.6 ± 5.7	182.4 ± 7.1***

Results are given as mean ± SEM. Expression of mRNA is normalized to 18S rRNA and is multiplied by 10^7^. The data were analyzed by full three-factor ANOVA tests with strain, exposure and time as categorical variables. There were no triple interactions between the type of strain, exposure and time. ^a^:Due to uneven variance, we determined 95; 99 and 99.9% confidence interval for means. *, **, *** refer to statistical significance *p *< 0.05, *p *< 0.01 and *p *< 0.001, respectively. Interactions: *Mip-2*, Strain*Exposure p < 0.05; *Mcp-1*, Strain*time p < 0.05, Strain*Exposure p < 0.05; *Il-6*, Exposure*Time p < 0.05, Strain*Exposure p < 0.05; Macrophages%, Exposure*Time p < 0.001, Strain*Exposure p < 0.01; Protein, Exposure*Time p < 0.01, Strain*Exposure p < 0.001.

##### BAL fluid: Cell differentiation, genotoxicity, protein and LDH

No significant difference was detected in the neutrophil and macrophage fractions in either strain following 3 h. However, there was a tendency for an increased fraction of neutrophils in ApoE^-/- ^mice. The fraction of neutrophils continued and was significantly elevated following 24 h. The fraction of neutrophils was also statistically significant following 24 h in C57 mice, although less so than in ApoE^-/- ^mice. The concentration of protein in BAL fluid is a marker for vascular permeability and cellular damage within the lung. The level of protein in ApoE^-/- ^mice was significantly elevated at both time-points (1.4 and 1.6-fold). The BAL protein in C57 mice was decrease at 3 h, but this is likely a chance finding because of two very low samples in this group. Leakage of LDH is another measure of dead or membrane damaged cells. We did not detect any differences in LDH content of the BAL fluid (data not shown). We presume the dilution of LDH in BAL fluid is too large to detect possible differences. Overall the C57 strain responded weaker and slower to the exposure.

### Instillation and inhalation of CB in ApoE^-/- ^mice

To determine possible differences between the methods of pulmonary exposure, we exposed ApoE^-/- ^mice to two doses CB delivered by instillation or inhalation. All animals were killed following 24 h. Results are shown in Table 2.

**Table 2:** Expression (mRNA) of *Mip-2*, *Mcp-1 *and *Il-6 *in lung tissue and cell distribution and protein in BAL fluid 24 h after inhalation or instillation of carbon black in ApoE^-/- ^mice.

		**Control**	**Low dose**	**High dose**	**significant dose-related differences**^b^
**Inhalation**		**HEPA air 1/2–1 1/2 h**	**CB 60 mg/m^3^, 1/2 h**	**CB 60 mg/m^3^, 1 1/2 h**	
**lung tissue**	***Mip-2***	9.9 ± 1.6	11.6 ± 2.9	17.6 ± 3.1	High dose ≈ Low dose
	***Mcp-1***	44.4 ± 10.5	97.2 ± 24.8*	79.9 ± 18.7	Low dose ≈ High dose
	***Il-6***	3.4 ± 0.6	4.3 ± 0.9	3.2 ± 0.5	Low dose ≈ High dose
					
**BAL**	**Neutrophils%**^a^	1.1 ± 0.4	0.7 ± 0.3	5.6 ± 3.2	High dose ≈ Low dose
	**Macrophages%**^a^	97.5 ± 0.3	97.9 ± 0.2	92.5 ± 4.1	High dose ≈ Low dose
	**Total BAL cells**	54750 ± 4891	66567 ± 6304	77867 ± 4896	
	**Protein**	91.2 ± 3.7	108.1 ± 7.5*	118.5 ± 5.5**	High dose ≈ Low dose
					
**Instillation**		**Vehicle control**	**CB 18 μg**	**CB 54 μg**	
					
**lung tissue**	***Mip-2***	5.1 ± 0.5	37.1 ± 13.4***	134.8 ± 33.2***	High dose>>>Low dose
	***Mcp-1***	28.3 ± 2.7	511.2 ± 246.7***	1087 ± 310.6***	High dose ≈ Low dose
	***Il-6***	1.1 ± 0.1	14.3 ± 7.2***	44.0 ± 13.0***	High dose>Low dose
					
**BAL**	**Neutrophils%**^a^	5.3 ± 1.6	41.3 ± 10.2*	75.8 ± 3.4***	High dose ≈ Low dose
	**Macrophages%**^a^	93.6 ± 1.5	57.7 ± 10.2***	22.1 ± 3.7***	High dose>>>Low dose
	**Total BAL cells**	65290 ± 5246	88173 ± 19861	78596 ± 21414	
	**Protein**	110.6 ± 5.7	150.4 ± 7.7***	182.4 ± 7.1***	High dose>Low dose

Results are given as mean ± SEM. Expression of mRNA is normalized to 18S rRNA and is multiplied by 10^7^. The effect of exposure was tested by nested ANOVA tests with the dose of CB nested in the method of exposure. Normal distributions of the residuals of nested ANOVA tests were assessed by the Kolmogorov-Smirnov tests with 5% as significance level. ^a^:Due to uneven variance, we determined 95; 99 and 99.9% confidence interval for means. ^b ^≈ , >, >>, >>> indicate statistical significance *p *> 0.05, *p *< 0.05 *p *< 0.01 and *p *< 0.001, respectively. *, **, *** refer to statistical significance *p *< 0.05, *p *< 0.01 and *p *< 0.001, respectively. Statistical difference by method of exposure: *Mip-2*, *p *≈ 0.08; *Mcp-1*, *p *< 0.001; *Il-6*, *p *< 0.05; Neutrophils%, *p *< 0.01; macrophages%, *p *< 0.001; Protein, *p *< 0.001;

#### Lung tissue: Expression of cytokine mRNA

Instillation of CB produced stronger effects on the mRNA levels of *Mcp-1 *and *Il-6 *compared to inhalation (p < 0.05 and p < 0.001, nested AVOVA, respectively). Inhalation of CB caused marginal increases in cytokine mRNA levels between 1.2- to 2.1-fold and 0.9- to 1.8-fold at low and high dose, respectively. Only *Mip-2 *at the low dose was significantly increased. In contrast instillation of the high dose of CB caused a greater response than the low dose. Instillation of low and high doses of CB resulted in 7- to 18-fold and 26- to 40-fold increase in cytokine mRNA levels, respectively.

#### BAL fluid: Cellular composition, protein concentration and LDH

Whether the exposures were by inhalation or instillation was a significant predictor of cellular composition and protein concentration in the BAL fluid (p < 0.01 (% of neutrophils), p < 0.001 (% of macrophages), p < 0.001 (protein concentration), nested ANOVA, respectively). The inhalation of CB was only associated with a marginally altered distribution between neutrophils and macrophages, whereas the i.t. instillation dose-dependently shifted the distribution towards increased representation of neutrophils in the BAL fluid. The concentration of protein was significantly elevated following inhalation and instillation at both doses. Inhalation of CB resulted in 108.1 and 118.5 μg protein/ml BAL fluid (high/low dose, respectively) compared to 91.2 μg/ml for the controls. This corresponds to a 1.2- and 1.3-fold induction, respectively. In comparison, the i.t. instillation of CB was associated with markedly larger concentration of protein in the BAL fluid of both the low and high dose of CB (1.4- and 1.7-fold, respectively). We did not detect any differences in LDH content of the BAL fluid when CB inhalations were compared to HEPA air inhalation or CB instillations were compared to control instillations (data not shown).

### Instillation of Au, C_60_, SWCNT and CB in ApoE^-/- ^mice

The inflammatory potential and ability of inflicting lung cell injury (i.e. protein concentration in BAL fluid) of Au, C_60_, SWCNT and CB was assessed 3 and 24 h following instillation by several end points in ApoE^-/- ^mice. In addition, the level of genotoxicity in BAL was assessed as a sensitive marker of early pulmonary toxicity.

#### Lung tissue: Expression of cytokine mRNA

As shown in Table 3, there were highly significant increases of *Mip-2*, *Mcp-1 *and *Il-6 *mRNAs in response to SWCNT and CB instillation at both time points. Overall, these two particles were by far more potent at the three end points when compared to Au and C_60_. SWCNT elicited the highest response at 3 h after instillation (52 – 195-fold) whereas CB increased the response 11 – 26-fold. Twenty-four hours following instillation the order was reversed. CB instilled mice showed increased cytokine levels between 26- and 40-fold whereas SWCNT instilled mice showed increased cytokine levels of 7- to 30-fold. Au and C_60 _particles significantly increased *Mcp-1 *following 3 h but to a much lesser than CB and SWCNT. Au also elicited a small but significant increase in *Mip-2 *response following 3 h. However this increase was due to a single outlier. The outlier increased the mean and SEM from 17.3 ± 4.1 up to 34 ± 17.1 and caused the statistical significance. Following 3 h no response was seen in *Mip-2 *following C_60 _instillation or in *Il-6 *with either particle. Au particles did not increase any cytokine end-point following 24 h whereas C_60 _significantly increased all compared to controls (4–6-fold). The increase however was weaker compared to SWCNT and CB.

**Table 3:** Expression (mRNA) of *Mip-2*, *Mcp-1 *and *Il-6 *in lung tissue and cell distribution, DNA damage by the comet assay and protein in BAL fluid 3 and 24 h after instillation of Au and carbonaceous nanoparticles in ApoE-/- mice.

		**Control**	**Au 0.54 μg**	**C**_60_**54 μg**	**SWCNT 54 μg**	**CB 54 μg**	**significant dose-related differences**^b^
**3 h**							
**lung tissue**	***Mip-2***	10.2 ± 3.2	34.0 ± 17.1*	10.9 ± 2.0	1170.9 ± 530.1***	108.1 ± 15.1***	SWCNT ≈ CB>Au>C_60_
	***Mcp-1***	10.1 ± 1.2	20.4 ± 3.4***	20.4 ± 2.1***	526.7 ± 214.8***	265.5 ± 163.9***	SWCNT ≈ CB>>>C_60 _≈ Au
	***Il-6***	2.1 ± 0.4	2.7 ± 0.7	1.7 ± 0.2	411.5 ± 180.6***	31.4 ± 4.8***	SWCNT>>>CB>>>Au ≈ C_60_
							
**BAL**	**Neutrophils %**^a^	3.7 ± 1.2	5.5 ± 2.6	2.8 ± 0.7	19.1 ± 8.9	13.8 ± 10.9	SWCNT ≈ CB ≈ Au ≈ C_60_
	**Macrophages %**^a^	93.9 ± 2.0	93.8 ± 2.5	96.9 ± 0.7	78.8 ± 9.0	83.1 ± 10.7	SWCNT ≈ CB ≈ Au ≈ C_60_
	**Total BAL cells**	83262 ± 4819	65081 ± 8276	68929 ± 3849	55426 ± 16930	49417 ± 7700	
	**Comet %T**	9.6 ± 0.6	11.6 ± 0.9	12.1 ± 0.9	13.4 ± 1.3**	14.4 ± 1.6**	CB ≈ SWCNT>>C_60 _≈ Au
	**Protein μg/ml**	102.5 ± 5.2	105.2 ± 6.2	77.4 ± 2.9**	171.8 ± 22.2***	139.3 ± 17.3**	SWCNT ≈ CB>Au>>C_60_
							
**24 h**							
**lung tissue**	***Mip-2***	5.1 ± 0.5	8.3 ± 1.7	31.0 ± 12.8***	34.4 ± 9.0***	134.8 ± 33.2***	CB>SWCNT ≈ C_60_>>Au
	***Mcp-1***	28.3 ± 2.7	34.6 ± 8.5	116.0 ± 22.8***	372.7 ± 110.2***	1087.0 ± 310.6***	CB ≈ SWCNT>>C_60_>>>Au
	***Il-6***	1.1 ± 0.1	2.0 ± 0.5	5.6 ± 1.1***	32.5 ± 9.9***	44.0 ± 13.0***	CB ≈ SWCNT>>C_60_>>Au
							
**BAL**	**Neutrophils %**^a^	5.3 ± 1.6	4.2 ± 3.1	6.4 ± 4.2	64.7 ± 7.1***	75.8 ± 3.4***	CB ≈ SWCNT>>>C_60 _≈ Au
	**Macrophages %**^a^	93.6 ± 1.5	94.7 ± 3.6	93.1 ± 4.2	28.6 ± 4.9***	22.1 ± 3.7***	CB ≈ SWCNT>>>C_60 _≈ Au
	**Total BAL cells**	65290 ± 5246	67881 ± 6667	76008 ± 7420	61643 ± 19999	78596 ± 21414	
	**Protein μg/ml**	110.6 ± 5.7	109.5 ± 5.2	83.6 ± 7.8***	288.8 ± 21.3***	182.4 ± 7.1***	SWCNT>>>CB>>>Au>>>C_60_

Results are given as mean ± SEM. Expression of mRNA is normalized to 18S rRNA and is multiplied by 10^7^. The statistical analysis was carried out with one-factor ANOVA tests and the type of particles as categorical variable. ^a^:Due to uneven variance, we determined 95; 99 and 99.9% confidence interval for means. ^b ^≈ , >, >>, >>> indicate statistical significance *p *> 0.05, *p *< 0.05, *p *< 0.01 and *p *< 0.001, respectively. *, **, *** refer to statistical significance *p *< 0.05, *p *< 0.01 and *p *< 0.001, respectively.

#### BAL fluid: Cell differentiation, genotoxicity, protein and LDH

Increased levels of neutrophils and decreased levels of macrophages were detected at both time points following exposure for CB and SWCNT. However, the altered cell composition was only significant following 24 h. Au and C_60 _instillations did not result in statistically different cell composition at any time point. The comet assay was used for determining DNA damage in BAL cells. BAL cells obtained 3 h after CB and SWCNT instillation, but not following Au and C_60 _instillation, had elevated level of DNA damage measured as % DNA in the tail. When we analysed the data by tail length, all four particles induced significant DNA damage (*p *< 0.001). SWCNT and CB exposure significantly increased the amount of protein in BAL fluid at both 3 h (1.7 and 1.4-fold, respectively) and 24 h (2.6 and 1.6-fold, respectively). Unexpectedly, the exposure for C_60 _caused a significant decrease in measured protein in BAL fluid. Since this was visible at both time points, it may be a genuine effect on the lung by C_60 _or it may be caused by C_60 _assay interference. Au exposure did not alter level of protein in BAL fluid. We did not detect any differences in LDH content of the BAL fluid (data not shown).

### Instillation of QD620 and QD621 in ApoE^-/- ^mice

Positively (QD621) and negatively (QD620) charged QDs were instilled in ApoE^-/- ^mice, to evaluate inflammatory potential of QDs as well as altered response caused by surface charge. Unlike the other particles and the QD vehicle, the QD instillation strongly affected the behavior the mice. We observed signs of apathy, piloerection and general discomfort. A microscopic examination revealed that 24 h after QD-instillation, the mice had developed acute pulmonary inflammation with edema and beginning hepatic necrosis. There was no sign of apoptosis in the liver by the TUNEL-assay and there were no changes in the kidneys. The QD-vehicle controls were unaffected.

#### Lung tissue: Expression of cytokine mRNA

As shown in Table 4, instillation of both QDs caused a highly significant increase in the level of all three cytokine mRNAs at both time points. The increase compared to vehicle was 10 – 25-fold at 3 h and 25 – 250-fold at 24 h. The positively charged particle (QD621) was more potent at all cytokine measurements (1.1 – 2-fold).

**Table 4:** Expression (RNA) of *Mip-2*, *Mcp-1 *and *Il-6 *in lung tissue and cell distribution, DNA damage by the comet assay and protein in BAL fluid 3 and 24 h instillation of negatively (QD620) and positively (QD621) charged CdTe quantum dots in ApoE-/- mice.

		**Vehicle**	**QD620**	**QD621**	**significant dose-related differences**^b^
**3 h**					
**lung tissue**	***Mip-2***^a^	36.0 ± 8.0	355.9 ± 38.3***	488.6 ± 188.4***	QD621 ≈ QD620
	***Mcp-1***	20.4 ± 2.7	246.2 ± 61.7***	416.6 ± 127.6***	QD621 ≈ QD620
	***Il-6***	13.3 ± 0.8	172.0 ± 30.4***	341.0 ± 97.3***	QD621 ≈ QD620
					
**BAL**	**Neutrophils %**^a^	6.8 ± 2.2	11.1 ± 6.0	10.8 ± 4.2	QD620 ≈ QD621
	**Macrophages %**^a^	91.8 ± 2.1	87.6 ± 6.2	86.4 ± 5.0	QD620 ≈ QD621
	**Total BAL cells**	54120 ± 6853	28965 ± 3017	34034 ± 3195	
	**Comet %T**	9.0 ± 1.1	29.3 ± 3.3***	29.7 ± 2.9***	QD621 ≈ QD620
	**Protein**	131.2 ± 8.6	129.4 ± 4.0	153.2 ± 10.5	QD621 ≈ QD620
					
**24 h**					
**lung tissue**	***Mip-2***	33.4 ± 14.4	848.4 ± 205.1*	920.7 ± 148.8**	QD621 ≈ QD620
	***Mcp-1***	152.2 ± 43.5	4471.9 ± 1613***	5956.3 ± 817.5***	QD621 ≈ QD620
	***Il-6***	6.5 ± 1.6	1114 ± 471.6***	1626.7 ± 531.6***	QD621 ≈ QD620
					
**BAL**	**Neutrophils %**^a^	20.6 ± 9.1	93.2 ± 2.1***	97.1 ± 0.7***	QD621 ≈ QD620
	**Macrophages %**^a^	73.3 ± 8.3	5.7 ± 1.4**	2.6 ± 0.6**	QD621 ≈ QD620
	**Total BAL cells**	63360 ± 7420	199600 ± 44198	308000 ± 101621	
	**Protein**	223.4 ± 29.6	560 ± 32.8***	675.8 ± 80.3***	QD621 ≈ QD620

Results are given as mean ± SEM. Expression of mRNA is normalized to 18S rRNA and is multiplied by 10^7^. The statistical analysis was carried out with one-factor ANOVA tests and the type of particles as categorical variable. ^a^:Due to uneven variance, we determined 95; 99 and 99.9% confidence interval for means. ^b ^≈ indicate statistical significance *p *> 0.05. *, **, *** refer to statistical significance *p *< 0.05, *p *< 0.01 and *p *< 0.001, respectively.

#### BAL fluid: Cell differentiation, genotoxicity, protein and LDH

We detected a slight insignificant increase in neutrophils and accordingly decrease in macrophages following 3 h in BAL fluid. The cell composition was significantly altered following 24 h with 93 – 97% neutrophils compared to 20% in vehicles. BAL cells obtained 3 h after QD exposure contained significantly increased level of DNA damage (3.3-fold), whereas there was no difference in the level of DNA damage elicited by the two different types of QDs. In addition, the DNA damaging effect of QDs was larger than the other types of nanoparticles used in this paper. No significant changes were detected in leakage of protein in BAL fluid following 3 h, although QD621 does appear to have caused an insignificant increase. Both QDs caused a highly significant increase following 24 h. We detected a significant decrease in LDH levels following QD exposure. Additional tests suggest that cadmium inhibits the LDH assay causing the decrease (data not shown).

## Discussion

We here present results that show that particle instillation induced a faster and stronger lung inflammatory response in hyperlipidemic ApoE^-/- ^mice compared to wild-type mice. Instillation produced stronger effects than inhalation. SWCNT, CB, C_60 _and gold nanoparticles showed inflammatory effects corresponding to their surface areas after instillation, whereas QDs were highly toxic, possibly due to cadmium leakage.

For determining the ranking order of the inflammatory potential of the particles, we intended to evoke a substantial pulmonary inflammation using a susceptible animal model. The exposure dose, 54 μg per mouse, is relatively large, but is well within the range used by others within the field of nanotoxicology [30-32]. Recently, hyperlipidemic ApoE^-/- ^mice have been used extensively to study the mechanisms involved in the vascular and particularly the atherothrombosis-inducing effects of exposure to both air pollution particles and SWCNT [33,4]. These studies have shown that such exposure accelerates vascular plaque formation [34,33,35] as well as cause hepatic oxidative stress and endothelial dysfunction even in 10–13 weeks old ApoE^-/- ^mice with less than 1% plaques in the aortic wall [36-38]. Although, ApoE^-/- ^and C57 mice responded to CB instillation, the response was much stronger in the former group particularly 3 h after exposure. This may indicate that ApoE^-/- ^are more sensitive or are primed to a response. The elevated susceptibility of the ApoE^-/- ^mice is important because these mice at young age display a modest dyslipidemia with elevated levels of cholesterol in plasma. Thus, they can be used as a experimental model because a large proportion of the human population in the Western World has elevated levels of lipids in the blood. In this respect it is interesting that the blood vessels in ApoE^-/- ^mice have increased permeability, and that ApoE^-/- ^mice have greater leakage of particulates across the blood-brain-barrier [18-20]. Probably the different lipid composition the cell membranes of ApoE^-/- ^mice affects permeability. It might be that the alveolar-blood barrier of ApoE^-/- ^mice also is more permeable to nanoparticles, or that inflammatory target cells are more permeable. Nevertheless, the recognition of an increased lung inflammation response is important for the interpretation of enhanced systemic effects in ApoE^-/- ^mice following pulmonary exposure. Especially because the suggested mechanisms of action for the atherothrombotic effects of nanoparticles include events secondary to pulmonary inflammation [4,5]. At present we are investigating the relationship of the pulmonary effects with cardiovascular effects in ApoE^-/- ^mice with some of the nanoparticles (data not shown).

In the past, there has been tremendous work done on comparisons of the pulmonary effect elicited by either instillation or inhalation (REFERENCES), whereas such comparisons are sparse in transgenic models representing susceptible human populations. We compared the lung inflammation elicited by instillation and inhalation of CB in presumed similar doses in ApoE^-/-^mice. We instilled 18 μg and 54 μg and this is almost identical to the pulmonary deposited doses (17.6 and 52.7 μg) estimated by assuming 33.8% deposition of the mass during a CB mouse inhalation exposure. Despite the apparently similar deposited dose, inhalation of CB caused much less inflammatory response than instillation did. None of the cytokine markers increased significantly following inhalation (up to 2.1-fold), whereas all were significantly increased following instillation (up to 40-fold). The fraction of neutrophils in BAL cells reached 6% following inhalation and 76% following instillation. Only the protein content of BAL fluid increased significantly and in a dose dependent manner following inhalation. However, protein levels were still less than for instilled animals. This difference between i.t. instillation and inhalation exposure is in keeping with the general conclusion from the literature that the clearance of instilled particles from the lung is slower and inflammation is greater (reviewed by [39]).

We have earlier studied pulmonary inflammation after inhalation of CB and diesel exhaust particles [40,26,43]. Inhalation is the "gold standard" for determining the potential toxicity of inhalants. It is the closest to a normal route of entry and the distribution pattern may also correspond more closely to that of a true exposure scenario with particles being deposited through the pulmonary system dependent on their size and shape. Advantages with instillation are the small and very precise amount of test material used and deposited. The benefits and problems of delivering materials to the lung by inhalation and instillation have been discussed in the literature (although available data are almost exclusively based on rats) [39,44,45]. Generally, intratracheal instillation is a well accepted procedure which usually well reproduces the effects of inhalation. However, the bolus administration by instillation may produce less homogeneous distribution of the material, with more focal exposure than following inhalation. Instillation also forces material into the alveoli resulting in lesser deposition in bronchia or bronchioles and may overwhelm mucociliary clearence.

By the cytokine mRNA levels, the inflammatory response in lung tissue was increased 52- to 195-fold following 3 h and 7- to 30-fold following 24 h after SWCNT instillations compared to controls. This indicates that the inflammation after SWCNT exposure is very strong, but that the primary inflammatory signalling ceases earlier, as has been suggested before [46,47,32]. CB also elicited a rapid (3 h) strong inflammation, although not as strong as SWCNT did. Interestingly, all markers of inflammation in lung tissue and BAL fluid continued to increase from 3 h to 24 h after the instillation. Li and co-workers found an influx of neutrophils following 6 and 24 h of more than 40-fold following a single 125-μg instillation dose in rats of CB. The neutrophil count was still elevated more than 10-fold after 7 days. Tumor necrosis factor-α activity measured *ex vivo *in BAL leukocytes from the above mentioned CB exposed rats continued to increase through the 7 days [48], suggesting a longer inflammation than SWCNT. C_60 _overall caused much less inflammation, with only *Mcp-1 *increasing significantly after 3 h (2-fold). The mRNA levels increased from 3 to 24 h with 4- to 6-fold significant elevation at 24 h. In all but one study [49] C_60 _was not toxic in rodents [50-55], even following intra-peritoneal delivery of up to 5 g/kg [52]. Although C_60 _was the least toxic of the carbonaceous particles, there may be a rapid distribution across membranes if agglomerates disintegrate to the small primary size [56]. In general, instillation of gold particles caused a very low and transient inflammatory response by detectable increases in *Mip-2 *and *Mcp-1*. It should be noted that the mass of instilled gold particles was very low (0.54 μg) and it is not possible to estimate the effect of an instillation of 54 μg. However, because of the very small size of these particles, gold still had a high number concentration in the instilled fluid (0.675 × 10^13^/50 μl).

**Figure 3:**
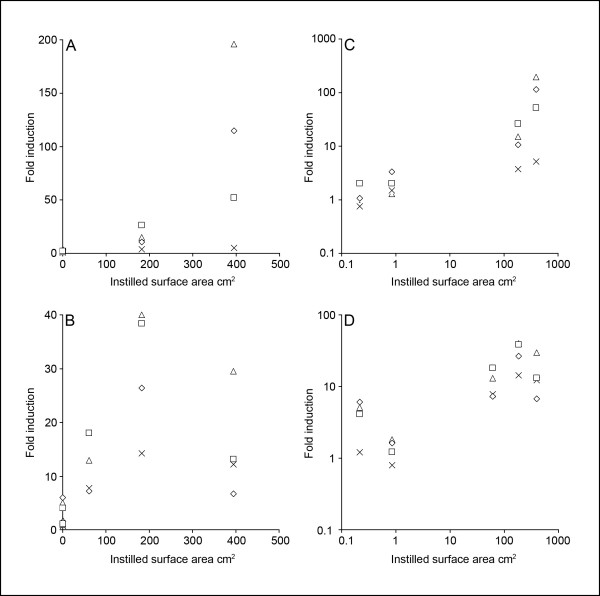
**Correlation between instilled surface area (vehicle, 0.54 μg gold, 54 μg C_60_, 54 μg CB and 54 μg SWCNT) and the fold induction on linear (A, B) and log scale (C, D) of each of the four inflammatory markers 3 h (A, C) and 24 h (B, D) after instillation**. 24 h figure additionally includes data from 18 μg CB. Figure legends and correlation coefficients (r^2^) at 3 h are: Squares, *Mcp-1 *= 1.00; diamonds, *Mip-2*, 0.85; cross, neutrophils = 0.94; triangles, *Il-6 *= 0.84; At 24 h r^2^-values were below 0.60 for all four datasets.

It has been shown previously that the inflammatory response of low toxicity-low solubility particles is proportional to the surface area of the instilled particles rather than the mass [57-60]. We have previously determined surface area of SWCNT, CB and C_60 _by the BET N_2 _adsorption method to be 731, 338 and <20 m^2^/g [61], indicating that we have instilled 395, 183 and 11 cm^2 ^of these three particles, respectively. The BET surface area of the C_60 _sample has since been corrected to 0.4 m^2^/g using a custom made single point BET modified for low surface areas (*Personal communication Giulio Pojana*) indicating that we have instilled 0.2 cm^2^. We also calculated the surface area of the instilled gold to be 0.85 cm^2^. The relationship between the surface area of the three carbonaceous particles (also 18 μg CB at 24 h), gold and controls and induction of cytokine mRNA and increased neutrophil fraction is illustrated in Figure 3. There was a good correlation between mRNA induction and fraction neutrophils at 3 h and the surface area instilled (r^2 ^between 0.84 and 1). However, the mRNA levels decreased between 3 h and 24 h after SWCNT instillation and there was a smaller fraction of neutrophils compared to CB instilled mice. Thus, SWCNT was less inflammatory following 24 h than anticipated from surface area alone. This means that r^2^-values were below 0.60 for all inflammatory end-points. It should be noted that nanoparticles were delivered as agglomerates and the factual instilled surface area may therefore be lower than estimated from BET. But that it has also been suggested that action of surfactants and proteins in the lung may reduce van der Waals interactions between nanoparticles and disperse these agglomerates [46].

Recently, it was suggested that there is threshold for inflammatory effects of low-toxicity, low-solubility particles at 1 cm^2 ^deposited particle surface area/cm^2 ^epithelial surface in the proximal alveolar region of the lung [62]. If the particle burden within a day is concentrated to the proximal alveolar region, the effect on interleukin expression after 24 h (but not after 3 h) might be interpreted as a threshold at between 1 and 30 cm^2 ^particle surface area per mouse (figure 3D). If we assume that the mice had lung has a epithelial surface area of about 600 cm^2 ^and 5% of this was in the proximal alveolar region [62] figure 3D might be interpreted as showing a threshold between 0,03 and 1 cm^2 ^deposited particle surface area/cm^2 ^epithelial surface area in the proximal alveoli, which would be in concordance with the theory of Donaldson et al. However, more data are needed to confirm this.

Numerous physicochemical parameters have been suggested to influence the inflammatory effects nanoparticles, including agglomeration state, shape, composition, surface reactivity, radical formation capacity and more [63]. SWCNT have a needle like shape and strong tendency to agglomerate. These parameters may distinguish SWCNT from C_60 _and CB and provide a possible explanation for the detected differences following 24 h. On the other hand, we have also previously found that the CB employed here caused more reactive oxygen species (ROS) formation than the SWCNT in cellular and cell-free system, whereas C_60 _generated far less ROS than the other particles [61]. Additionally, recent evidence may suggest that SWCNT produces long term toxicity and may be different from low toxicity particles [30,46,47,64,32]. The BAL protein at 24 h showed the strongest correlation with surface area (r^2 ^= 0.98 and 0.86 at 3 h)(not shown).

Since the QDs required thioglycolic acid as stabilizer, we exposed mice in the control group to a vehicle solution that contained this chemical. It is evident from the results that adding thioglycolic acid to the vehicle causes an inflammatory response with 2- to 6.5-fold increased mRNA cytokine levels after 3 h and 24 h, where the fraction of neutrophils was increased 4-fold. The pulmonary inflammation was quite severe following the instillation of QDs with increases of 25–250-fold of the cytokine mRNA levels over the vehicle control. Almost all cells (93–97%) in the BAL fluid were neutrophils at 24 h. However, there was a remarkably similar magnitude of the inflammatory response of both negatively and positively charged QDs. We are not aware of other *in vivo *studies on cytokine induction by QD exposure. However it has been shown that several different cytokines, including IL-6 and MCP-1 are induced 2- to 3-fold in human epidermal keratinocytes and rat mesenchymal stem cells following QD exposure [65,66]. The CdSe QDs used with the cells were coated with ZnS shell, which probably decreases the toxicity. Similarly mice have been injected i.v. with CdSe QDs, but no noticeable adverse effects or signs of necrosis at sites of deposition were reported [13,67]. It therefore appears likely that cadmium was leaking from the QDs that we tested. Indeed, Pearson and co-workers measured BAL protein following instillation of different concentrations of Cd^2+ ^in Male CF-1 mice. If we expect effects of the same magnitude this would mean that 18–26% of the cadmium in the QD620 and QD621 delivered to the mice might have been dissolved [68]. However, this will be overestimated if intact QDs or free telluride also show toxicity.

We determined the level of DNA damage by the comet assay in BAL cells 3 h following pulmonary exposure of all particles. At this time point the fraction of neutrophils was small and the BAL fluid primarily contained macrophages. The DNA damage increased significantly following exposure to SWCNT, CB and QDs, but only marginally with C_60 _and gold nanoparticles. QDs were also much more genotoxic than the other particles with a more than 3-fold increase in the level of DNA damage. Even when compared to the genotoxic carbonaceous particles (SWCNT and CB) we detected about 2-fold more DNA damage in the BAL cells. This may be a result of severe oxidative stress induced by cadmium leaked from the QDs. Indeed cadmium has been shown to induce oxidative stress with DNA damage and impair repair in several cell types [69]. The comet assay is considered to be a sensitive assay for detection of the genotoxic effect of particulate matter as has been shown by several studies on air pollution particles [70,43]. DNA damage is an important mechanism in carcinogenesis but the quantitative relationship of DNA damage and the development of cancer are not clear and it is probably different for different chemicals and for different cell populations. In this study we investigated DNA damage in BAL cells and not lung tissue, where lung disease would normally arise. We believe that the effects observed in BAL cells are similar in the epithelial cell of the lung surface because they are located closely together. However, detecting effects in lung tissue is difficult because only a fraction of cells in the lung tissue is exposed and the effects are diluted within the cell population. We have previously shown that CB exposure increased the level of strand breaks as well as mutation frequency in a mouse lung epithelial cell line [71]. We suspect a high ROS production to be the cause of these effects as repeatedly suggested [72-74].

## Conclusion

Here we report that ApoE^-/- ^mice is a sensitive model for comparing inflammatory potential of (nano) particle instillation. CB and SWCNT caused more inflammation and DNA and cell damage than C_60 _did. This inflammatory signalling appeared shorter for SWCNT than for CB and C_60_. The instilled surface area of low toxicity low solubility particles appears to be a good predictor for inflammatory response *in vivo*. QDs were the most toxic particle likely because of a Cd effect. Gold particles, at a lower dose than the other particles, did not induce an inflammatory or toxic response in the mice.

## Methods

### Mice and caging conditions

Female wild-type C57BL/6 (C57) and C57BL/6-Apoe^tm1 ^(ApoE^-/-^) mice aged 4–6 weeks were obtained from Taconic (Ry, Denmark). The mice were randomly divided into groups of 10 housed in polypropylene cages (425 mm × 266 mm × 150 mm) with pinewood sawdust bedding and enrichment as sticks of aspen wood and rodent tunnels (Brogaarden, Denmark). The cages were stored in rooms with a 12 h light period from 6 a.m. to 6 p.m., and the temperature and relative humidity in the animal room were 21 ± 2°C and 50 ± 5%, respectively. The cages were sanitized twice weekly. All mice were given free access to tap water and standard mouse chow diet (Altromin no. 1324, Christian Petersen, Denmark). The mice were kept under pathogen-limited conditions and were allowed to acclimatize for 2–4 weeks before they entered the experimental protocol. All mice were 8 weeks old at the time of the experiment. A total of 169 mice were used in this study, of which 141 were ApoE^-/- ^and 28 were C57 mice. After completing the experiments we were informed by the supplier that some of the animals might be heterozygous for the ApoE locus. All animals were genotyped and 12 of the 141 ApoE^-/- ^mice were found to heterozygotes. We have retained the data from these mice in the dataset for two reasons: The data from the heterozygous mice were not different from the homozygous ApoE^-/- ^mice and the experimental setup was designed to minimize the effect of day-to-day experimental variation in the exposure by having mice in different groups being exposed at the same day. All animal procedures followed the guidelines for the care and handling of laboratory animals established by the Danish government, and the Animal Experiment Inspectorate under the Ministry of Justice, approved the study.

### Study design

The study design is summarized in Table 5. The experiment consisted of four parts (Part 1–4) that were carried out in a general design with some exposure groups serving in more than one part in order to reduce the number of required animals. In Part 1 we compared the pulmonary toxicity 3 and 24 h following instillation of 54 μg CB or vehicle in C57 (groups of 7) and ApoE^-/- ^mice (groups of 7 for CB or 15 for vehicle). The results from the ApoE^-/- ^mice were also incorporated in the design and statistical analysis of part 2 and 3. Part 2 focused on determining whether the method of exposure caused differences in pulmonary response in ApoE^-/- ^mice. CB was either instilled (18 μg in n = 5 or 54 μg in part 1) or inhaled (60 mg/m^3 ^for 30 min (n = 5) or 90 min (n = 5). Control animals either had vehicle instilled or inhaled HEPA filtered air for either 30 min (n = 5) or 90 min (n = 5). All animals were sacrificed following 24 h. Part 3 assessed toxicity 3 and 24 h following instillation of vehicle, 0.54 μg gold or 54 μg of C_60_, SWCNT or CB with 7 ApoE^-/- ^mice in each exposure group and time point. Part 4 assessed toxicity 3 h and 24 h following instillation of vehicle QD621 or QD620 with 5 ApoE^-/- ^mice in each exposure group and time point. Mice were given 137.5 μg of QDs containing 63 μg of Cd.

**Table 5:** Experimental setup; including exposure, exposure dose, time of sacrifice and the number and strain of mice.

**Exposure**	**Vehicle** **Instillation**		**Au 0.54 μg** **Instillation**		**C_60 _54 μg** **Instillation**		**SWCNT 54 μg** **Instillation**		**CB 54 μg** **Instillation**		**CB 18 μg** **Instillation**	
**Time of sacrifice**	3 h	24 h	3 h	24 h	3 h	24 h	3 h	24 h	3 h	24 h		24 h
**# of C57 mice**	7 ^1^	7 ^1^							7 ^1^	7 ^1^		
**# of ApoE^-/- ^mice**	15 ^1,2,3^	15 ^1,2,3^	7 ^3^	7 ^3^	7 ^3^	7 ^3^	7 ^3^	7 ^3^	7 ^1,2,3^	7^1,2,3^		5 ^2^
**Exposure**	**Vehicle** **Instillation**		**QD620 63 μg Cd** **Instillation**		**QD621 63 μg Cd** **Instillation**		**HEPA inhalation 30 or 90 min**		**CB inhalation** **60 mg/m**^3^**30 min**		**CB inhalation** **60 mg/m**^3^**90 min**	
**Time of sacrifice**	3 h	24 h	3 h	24 h	3 h	24 h		24 h		24 h		24 h
**# of ApoE^-/- ^mice**	5 ^4^	5 ^4^	5 ^4^	5 ^4^	5 ^4^	5 ^4^		10 ^2^		5 ^2^		5 ^2^

All mice received exposure via instillation unless otherwise is stated in the table.

^1,2,3,4 ^Indicate the study part(s) the animals were included in (see design in Material and Methods section).

### Particles

The following materials were used in this study: CB, SWCNT, C_60_, gold and QD particles. The CB, Printex 90 was a gift from Degussa-Hüls, Frankfurt, Germany. The declared primary particle size is 14 nm. The EliCarb^® ^SWCNT was purchased as a dry powder from Thomas Swan and Co. Ltd. (Consett, UK). Declared primary particle size was 0.9–1.7 nm as diameter and ≤1 μm as length. The investigated C_60 _was 99.9% pure and were purchased through Sigma Aldrich, Denmark (Prod. 572500). The declared primary particle size was 0.7 nm. The three carbonaceous particles have previously been characterized with the following results. All results are listed as CB, SWCNT and C_60_, respectively. Brunauer, Emmett and Teller (BET) surface area (m2/g); 338, 731 and < 20. Average pore size (nm); 60, 15 and 0. Content of the 16 US-EPA priority polyaromatic hydrocarbons (ng/g); 75, 417 and 0. Declared carbon content (%); > 99%, ~95% and 99.9%. ICP-MS analysis revealed no contaminants in CB and C_60 _and low amounts in SWCNT (2% Fe, < 0.001% Co, Ni, Mn)[61]. The 2 nm gold particle solution contained 15 × 10^13 ^particles per 1 ml and a mass of 12.13 μg (Fitzgerald Industries International, USA). These particles were made by citrate reduction and therefore had a negative surface charge. The gold nanoparticles were monodisperse and spherical in shape. The solution additionally contained 0.01% AuCl and traces of citrate, pH = 5.5. Red emitting CdTe QDs 5.5 g/l (46% Cd and 29% Te) dispersed in water containing 5 ml/L or 0.0072 mol/L of thioglycolic acid as a stabilizer were purchased from American Dye Source Inc. . These QDs either had a positively charged (Cd-S-CH_2_-CH_2_-NH_3_^+ ^Cl^-^) or negatively charged (Cd-S-CH_2_-CH_2_-COO^- ^Na^+^)(ADS620QD) particle surface. The declared particle size was 4.5 – 5.5 nm.

### Preparation of exposure stocks

CB, C_60 _and SWCNT particles were suspended by sonication in 0.9% NaCl MilliQ water containing 10% v/v BAL from either C57BL/6 or ApoE^-/-^. The BAL fluid was prepared by flushing unexposed mice twice to 0.6 ml 0.9% NaCl yielding approximately 1 ml of BAL fluid. The CB, C_60 _and SWCNT particles (either 1.08 or 0.36 mg/ml) were sonicated using a Branson Sonifier S-450D (Branson Ultrasonics Corp., Danbury, CT, USA) equipped with a disruptor horn (Model number: 101-147-037). Total sonication time was 15 min, with alternating 55 s pulses and 5 s pauses at amplitude of 10%. Samples were continuously cooled on ice during the sonication procedure. Vehicle control solutions were prepared for C57 and ApoE^-/- ^mice containing 90% 0.9% NaCl MilliQ water and 10% BAL fluid from the appropriate strain and were sonicated as above. All solutions were divided into aliquots which were immediately frozen at -80°C. Gold suspensions were prepared as follows: On the morning of each gold instillation 100 μl BAL fluid from ApoE^-/- ^mice were thawed, and 900 μl gold solution and 8.1 mg NaCl was added. All solutions, freshly prepared gold or samples retrieved from the freezer, were stored on ice until used within a few hours. All QD exposures (3 h and 24 h) were conducted on the same day. The QD vehicle was prepared by mixing 800 μl MilliQ water, 5 μl thioglycolic acid (>99%), 8.1 mg NaCl, adjusting pH to 7.4 and then adding 100 μl BAL and up to 1 ml with MilliQ water. QDs (500 μl of either ADS620QD or ADS621QD) were mixed with 2.5 μl thioglycolic acid, 300 μl MilliQ, 8.1 mg NaCl, adjusted pH to 7.4 and then adding 100 μl BAL and up to 1 ml with MilliQ water. The suspensions were used within a few hours but were not kept on ice.

### Exposure of mice

The study consists of two exposure methods: A single i.t. instillation exposure or a single inhalation exposure. The doses of each particle, period, number of mice and strain as well as exposure method are described in section "Study design". To eliminate day to day variation, 3–4 materials were instilled on each exposure day and each animal cage delivered mice to minimally 3 different exposures.

#### I.t. instillation

The mice were anesthetized using Hypnorm^® ^(fentanyl citrate 0.315 mg/ml and fluanisone 10 mg/ml from Janssen Pharma) and Dormicum^® ^(Midazolam 5 mg/mL from Roche). Both were mixed with equal vol. sterile water. A volume of 0.2 ml was injected subcutaneously in the neck of each mouse. The sedated mice were kept on 37°C heating plates. During instillation the mice were placed on their backs on a 40 degree slope. A diode light was placed touching the larynx. The tongue was pressed towards the lower jaw by a small spatula. The trachea was intubated using a 24 gauge BD Insyte catheter (Ref: 381212, Becton Dickinson, Denmark) with a shortened needle. The correct location of each intubation was tested by a small but highly sensitive pressure transducer developed by our laboratory in collaboration with John Frederiksen (FFE/P, Copenhagen, Denmark). The particle suspensions were rigorously mixed by pipetting immediately before instillation. A 50 μl suspension was instilled followed by 150 μl air with a 250 μl SGE glass syringe (250F-LT-GT, MicroLab, Aarhus, Denmark). The intubation catheter was removed and the mouse transferred to a vertical hanging position with the head up. This ensures that the delivered material is maintained in the lung and does not block the airways. After 5 to 10 min the mice were transferred to the 37°C heating plate until they recovered from anaesthesia. The deposition and distribution of instilled material was verified installing Evans blue, radioactive gold (18 nm) and QDs (data not shown).

### Charactrerization of exposure

#### Instillation

The hydrodynamic particle number and volume distribution of the particles in the exposure liquids were analyzed by photon correlation spectroscopy using a Dynamic Laser Scatter (DLS) Zetasizer nano ZS (Malvern Inc., UK.) as previously described [61]. Number and volume distributions were calculated by the DTS software using the viscosity for H_2_O and reference values or suggested refractive (R_i_) and absorption indices (R_s_) for the different particles. Data quality was analysed by evaluating the intensity correllelogram, cumulants fit and the distribution fit of the laser scattering intensity data.

#### Inhalation

Mice were exposed to either CB aerosol or HEPA filtered air in a nose-only inhalation chamber. The aerosol was generated using a microfeeder with dispersion nozzle (Fraunhofer Institut Toxikologie und Aerosolforschung, Hannover, Germany). The mass concentration of particles in the chamber was calibrated by sampling onto 0.5-μm Fluoropore™ membrane filters (Millipore, Billerica, MA), the number of large particles (0.75 to > 15 μm) was continuously measured using a Dust monitor (Grimm, 1.105, Ainring, Germany). The CB mass concentrations in the aerosol were measured each 15 minute and were narrowly around the target concentrations of 60 mg/m^3^. The mean ± SEM and median concentration was 61.1 ± 3.3 and 59.25 ± mg/m^3^, respectively. The mean ± SEM and median of particles above 1 μm was 285 000/L ± 27 000 and 236 000/L. A one-hour long aerosolization experiment was conducted to determine the aerosol number and mass size distribution of the CB in the animal exposure chamber. Fine particles were measured using a GRIMM Sequential (Stepping) Mobility Particle Sizer connected (SMPS) consisting of a Long Electrostatic Classifier (Model No. 5.521; Serial No. 5LP 10209) connected to a GRIMM Condensation Particle Counter (Model 5.400). Particles were neutralized using a 3.7 MBq Am-241 source (Model No. 5.521) after passing through two impactors with nominal d_50 _cut-points of 1,185 and 805 nm were mounted externally and internally in serial at the DMA inlet and thoroughly cleaned after each round of exposure. At the CB density (2.1 g/cm^3^), the lower impactor stage has a d_50 _at 532 nm, which is the reason for 500 nm being the coarsest particles measured with the SMPS. Data sampling and calculations were completed using the GRIMM software 5.477/02 v. 1.34 in the fast scan mode, which performs a full size distribution analysis from 9.8 to 874.8 nm in 3 min and 38 sec. Data were corrected for both Classifier and CPC efficiency by the software. Coarse particles were measured using a GRIMM Dust Monitor at a resolution of 6 sec. The Dust Monitor particle sizes were subsequently recalculated to geometric means assuming an upper channel cut-point at 20 μm.

### BAL and isolation of organs

3 or 24 h after instillation or inhalation exposure, the mice were anaesthetised with Hypnorm/Dormicum as described above. To obtain BAL cells, the lungs were infused four times with 0.8 ml sterile 0.9% NaCl through the trachea. The BAL fluid was stored on ice until centrifugation at 400 × *g *for 10 min. The supernatant was stored at -80°C for Protein (Pierce BCA, Bie-Berntsen, Denmark) and LDH (Roche, Denmark) according to the manufacturer's protocols. The cell pellet was treated as described in [40] for determination of cell composition and cell storage for comet analysis. We found that almost all cells were either macrophages or neutrophils and the very small number of lymphocytes and eosinophils detected throughout all experiments were disregarded. Therefore macrophages and neutrophils will not always add up to 100%. Total cells counts could not be determined reliably and large variations were observed. After BAL isolation the lungs were quick-frozen in liquid nitrogen and stored at -80°C until further analysis.

### Comet analysis on BAL cells

The comet assay was as described in [40] with the following modifications. The cell-agarose mixtures were cast onto a 100 mm × 85 mm GelBond film (Cambrex Bio Science, Rockland, ME, USA) with a polyethylene moulding form (100 mm × 75 mm × 10 mm) with eight holes (*d *= 19.5 mm). Multiple measures exist when using comet assay. Based on the recommendation from an in vivo comet assay workgroup we chose to present % tail DNA since this appeared to be most linearly related to exposure dose when using image analysis [75].

### Preparation of RNA and cDNA from lung tissue

RNA from the entire right lung of each mouse was prepared by lysing the tissue in 875 μl SV lysis buffer, while vigorously disrupting the sample with a Tissuelyser (Qiagen, Denmark) with a 5 mm stainless steel bead for 2 × 60 seconds. RNA was purified from 175 μl using Promegas SV total RNA isolation system according to the manufacturers' protocol. RNA was eluted by 2 × 50 μl DEPC water. cDNA was prepared from DNase treated RNA using TaqMan^® ^reverse transcription reagents (Applied Biosystems, USA) as described by the manufacturers protocol.

### Real time RT-PCR

The *Mip-2*, *Il-6 *and *Mcp-1 *gene expression was determined using real-time RT-PCR with 18S RNA as reference gene as described by [26]. However, *Il-6 *was detected on ABI Prism 7300 and *Mcp-1 *and *Mip-2 *were detected on ABI Prism 7500 (PE Biosystems, Foster City, CA, USA).

### Statistics

All datasets were analysed by parametric ANOVA tests. Homogeneity of the variance was tested by Levene's test. Initial assessments indicated that some of the endpoints only had homogeneity of variance between groups after singular or double log transformation. For statistical simplicity, we chose to transform all results by double log transformation. These endpoints were tested by parametric ANOVA tests at the 5% level. The Fisher least significant difference test was used for the post-hoc comparisons between groups. The results on the percentage of neutrophils and macrophages in BAL fluid still had uneven homogeneity of variance between groups after data transformation. For these endpoints, we determined the 95%, 99% and 99.9% confidence intervals for the means of each of the groups. Statistical significance was obtained if confidence interval did not overlap. In the first part of the investigation (assessment of the difference between wild type and ApoE^-/- ^mice) the data were analyzed by full three-factor ANOVA tests with strain, exposure and time as categorical variables. In the second part of the study (method of exposure), the effect of exposure was tested by nested ANOVA tests with the dose of CB nested in the method of exposure. The nested ANOVA design was used as a conservative test instead of the regular full ANOVA design, because we did not know for sure if the doses delivered by inhalation and instillation were the same. Normal distributions of the residuals of nested ANOVA tests were assessed by the Kolmogorov-Smirnov tests with 5% as significance level. The statistical analysis of the data in part three (CB, C_60_, SWCNT, and Au) and four (QDs) were carried out with one-factor ANOVA tests and the type of particles as categorical variable. The statistical analysis was performed in Statistica 2002 for Windows (StatSoft, Uppsala, Sweden).

## Abbreviations

BAL: Broncho-alveolar lavage; C_60_: fullerenes C_60_; CB: carbon black; *Il-6*: interleukin-6; LDH: lactate dehydrogenase; *Mip-2*: macrophage inflammatory protein-2; *Mcp-1*: macrophages/monocyte chemoattractant protein-1; ROS: reactive oxygen species; SWCNT: single walled carbon nanotubes; i.t.: intratracheal; QD: quantum dot

## Competing interests

The authors declare that they have no competing interests.

## Authors' contributions

All authors contributed to the idea and design of the study. NRJ carried out all exposures, toxicological analysis and drafted the manuscript. KAJ analysed particle size distribution and developed the deposition model. HWA and PM participated substantially in the inhalation exposure and the statistical analysis, respectively. OL conducted the histopathological analysis of organs following QD and vehicle exposure. All authors contributed, read and approved the final manuscript.
